# Harmonizing heterogeneous transcriptomics datasets for machine learning-based analysis to identify spaceflown murine liver-specific changes

**DOI:** 10.1038/s41526-024-00379-3

**Published:** 2024-06-11

**Authors:** Hari Ilangovan, Prachi Kothiyal, Katherine A. Hoadley, Robin Elgart, Greg Eley, Parastou Eslami

**Affiliations:** 1 Science Applications International Corporation (SAIC), Reston, VA 20190 USA; 2 Scimentis LLC, Statham, GA 30666 USA; 3 Department of Genetics, Computational Medicine Program, Lineberger Comprehensive Cancer Center, University of North Carolina at Chapel Hill, Chapel Hill, NC 27599 USA; 4 University of Houston, Houston, TX 77204 USA; 5 Universal Artificial Intelligence Inc, Boston, MA 02130 USA

**Keywords:** Computational biology and bioinformatics, Molecular biology, Mathematics and computing, Particle physics

## Abstract

NASA has employed high-throughput molecular assays to identify sub-cellular changes impacting human physiology during spaceflight. Machine learning (ML) methods hold the promise to improve our ability to identify important signals within highly dimensional molecular data. However, the inherent limitation of study subject numbers within a spaceflight mission minimizes the utility of ML approaches. To overcome the sample power limitations, data from multiple spaceflight missions must be aggregated while appropriately addressing intra- and inter-study variabilities. Here we describe an approach to log transform, scale and normalize data from six heterogeneous, mouse liver-derived transcriptomics datasets (*n*_total _= 137) which enabled ML-methods to classify spaceflown vs. ground control animals (AUC ≥ 0.87) while mitigating the variability from mission-of-origin. Concordance was found between liver-specific biological processes identified from harmonized ML-based analysis and study-by-study classical omics analysis. This work demonstrates the feasibility of applying ML methods on integrated, heterogeneous datasets of small sample size.

## Introduction

NASA’s long-duration deep space missions beyond low earth orbit (LEO) will expose astronauts to ionizing radiation and microgravity for durations longer than previously encountered by humans^1,2^. Characterization and mitigation of the adverse effects of prolonged spaceflight are critical for mission success as well as protecting the long-term health of the astronauts. The Rodent Research (RR) missions conducted since 2014 use rodent models flown to the International Space Station (ISS). These missions, originally designed to evaluate testing hardware, have evolved to study relevant biomarkers^3^, characterize the biological responses of different organs and systems to the space environment, and translate this knowledge from animal studies to inform risk characterization and countermeasure development^4^.

When possible, next-generation sequencing (NGS), such as RNA-seq, has been applied to samples collected from the RR missions and the data generated from these experiments have been processed and stored in the NASA Open Science Data Repository (OSDR)^5^. NGS analysis further expands the biological insights generated from the RR mission models. Specifically, these data enable interrogation of the impacts of spaceflight at the molecular level to support translation from animal models to the human as many of the same underlying mechanisms that impact health outcomes are conserved across species. A robust understanding of how changes in gene expression may correlate with changes in relevant phenotypes associated with spaceflight can provide critical information to identify biomarkers for health surveillance and strategic countermeasure deployment, and targets for tactical countermeasure development to mitigate health risks. NASA’s heavy reliance on animal studies to characterize the risks associated with space radiation specifically, necessitates the ability to appropriately and effectively translate animal data to humans. One of the organs of interest and studied in spaceflight missions is the liver. The liver plays a critical role in carbohydrate and lipid metabolism, as well as the processing of xenobiotic substances. Additionally, hepatic cancer is considered radiogenic as it has been shown to develop following exposure to ionizing radiation in both humans and animals^6,7^. Therefore, characterization of the effects of spaceflight on the liver is essential for understanding the potential health risks to astronauts. Decrements in liver function in-mission could impact nutrient uptake as well as medication efficacy—two major human health risks for spaceflight. The development of liver cancer can also severely impact astronaut long-term health and well-being given the 5-year survival is currently under twenty percent^8^. Spaceflight experiments, as well as those using analogs of spaceflight hazards like microgravity or radiation have demonstrated impacts on liver processes and metabolism which are associated with liver disease or the development of liver cancer^9–13^.

However, due to the space and resource constraints on the ISS, the number of rodents per experimental group and the associated power of detection for small effect sizes are limited^14,15^. It is possible to address this limitation by appropriately combining the data from different RR missions to increase experimental power. Comprehensive data harmonization followed by the application of machine learning (ML)-based approaches can be leveraged to support identification of relevant biological pathways present across multiple spaceflight datasets.

ML-based techniques have been tested on RNA-seq by identifying informative features and formulating classification problems^16^. However, these techniques have the benefit of using large datasets^17^ or using single-cell RNA-seq datasets^15^ with many samples from a given cell population, with more data homogeneity^18^. For spaceflight studies limited by small sample size as well as differences in subject age, sex, strain, and flight duration, existing ML methods do not address the challenges in data harmonization and training of models in the presence of heterogeneity across datasets due to laboratory batch effects, differences in sample extraction, sequencing library preparation methods and bioinformatics processing pipelines^19,20^.

Our study leverages existing data on murine livers available in NASA OSDR to develop and validate a robust methodology to first combine and harmonize data, second apply ML techniques to high dimensional RNA-Seq datasets across several experiments, and third extract meaningful biological interpretation on molecular and genetic levels. In this analysis, the data generated from several RR missions conducted on the ISS are harmonized. A pipeline for end-to-end feature selection and model training is tested on several ML classifiers. The classification problem is encoded to correctly determine a spaceflight sample based upon biomarkers available in RNA-Seq data. The goal of the classification problem is to identify genes predictive of spaceflight, investigate them using prescriptive methods such as ranking and feature importance, and identify robust biomarkers of spaceflight. This study is focused on harmonization of RNA-seq data across multiple datasets to develop a predictive ML-based spaceflight classification model. Thus, the methods, and data handling and interpretation processes developed in this study will allow more powerful generalizable analysis using next-generation data for future biological pathway definition and countermeasure development relevant to spaceflight.

## Results

### Pipeline overview

**Fig. 1:**
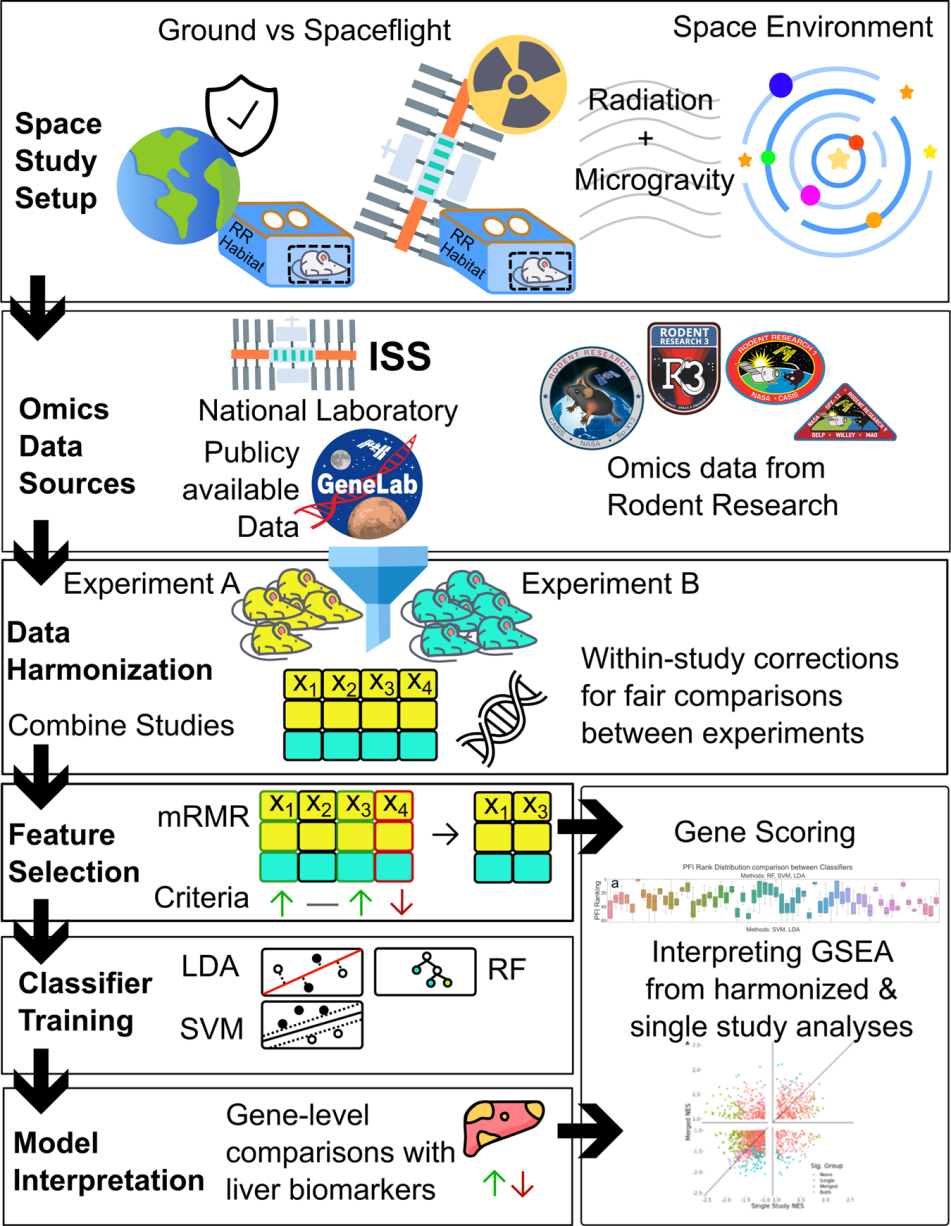
Process flowchart of end-to-end machine learning pipeline for rodent research spaceflight studies. Data generated from space studies typically include a ground control and spaceflown group that are exposed to the space environment, including galactic cosmic radiation and microgravity. For NASA Rodent Research missions, the spaceflown group is exposed to low Earth orbit via the ISS national laboratory. Groups of spaceflown mice are returned to earth, processed, and stored using standardized RNA-sequencing protocols at NASA Open Science Data Repository. Our pipeline harmonizes data across multiple rodent research missions through systematic preprocessing and dimensionality reduction using the mRMR criterion. Three classifiers, LDA, RF, and SVM are trained and interpretable at the gene-level using a standardized feature importance ranking. The harmonized data were compared with known liver biomarkers and classical differential gene expression analysis using GSEA. All NASA Rodent Research patches and the NASA GeneLab logo were sourced from NASA (nasa.gov). ISS International Space Station, mRMR minimum redundancy maximum relevance, RF Random Forest, SVM Support Vector Machine, LDA Linear Discriminant Analysis, GO gene ontology, BP Biological Processes, ML machine learning, GSEA gene set enrichment analysis.

The following results are from a supervised ML analysis of RNA-Seq data generated from murine livers across multiple spaceflight experiments. While per-study ML analysis is infeasible due to insufficient sample size, enough data are available across multiple studies to explore ML-based methods. However, integration of these datasets is complicated by differing sample size and cohort traits. This analysis addresses data heterogeneity and frames a ML classification problem that effectively distinguishes between spaceflown and ground control samples. The end-to-end pipeline shown in Fig. 1 breaks down key components of the proposed methodology from data selection, harmonization, feature selection, model training, and interpretation of results.

### Study Cohort

We identified six studies from five LEO NASA RR missions with murine liver RNA-Seq data. Data cleaning and outlier removal resulted in datasets ranging in size from 6-59 animals and displayed large heterogeneity due to biological factors including strain, age, and sex as well as technical variability associated with RNA-seq library preparation (Table 1).

**Table 1:** Study metadata and primary drivers of variability across murine liver datasets generated from Rodent Research missions

Study ID^a^	Mission^b^	Sample Size^c^	(SF | GC)	Strain	Age^d^ (wks)	Sex	Library Preparation^e^
47	RR-1 (CASIS)	6	(3|3)	C57BL/6NTac	32	F	mRNA enrichment
168	RR-1 (NASA)	10	(5|5)	C57BL/6J	16	F	Ribo-depletion
168	RR-3	8	(4|4)	BALB/c	12	F	Ribo-depletion
242	RR-9	18	(5|13)	C57BL/6J	10	M	Ribo-depletion
245	RR-6	36	(17|19)	C57BL/6NTac	32	F	Ribo-depletion
379	RR-8	59	(27|32)	BALB/cAnNTac	10-32	F	Ribo-depletion

*SF* spaceflown, *GC* ground control.

^a^GeneLab data set (GLDS) identifier.

^b^The Rodent Research (RR) mission identifier.

^c^The sample size in the spaceflown and ground control groups after removal of outliers and technical replicates.

^d^Age of rodent at launch.

^e^Protocol used during RNA sequencing.

### Prefiltering and feature selection

**Fig. 2:**
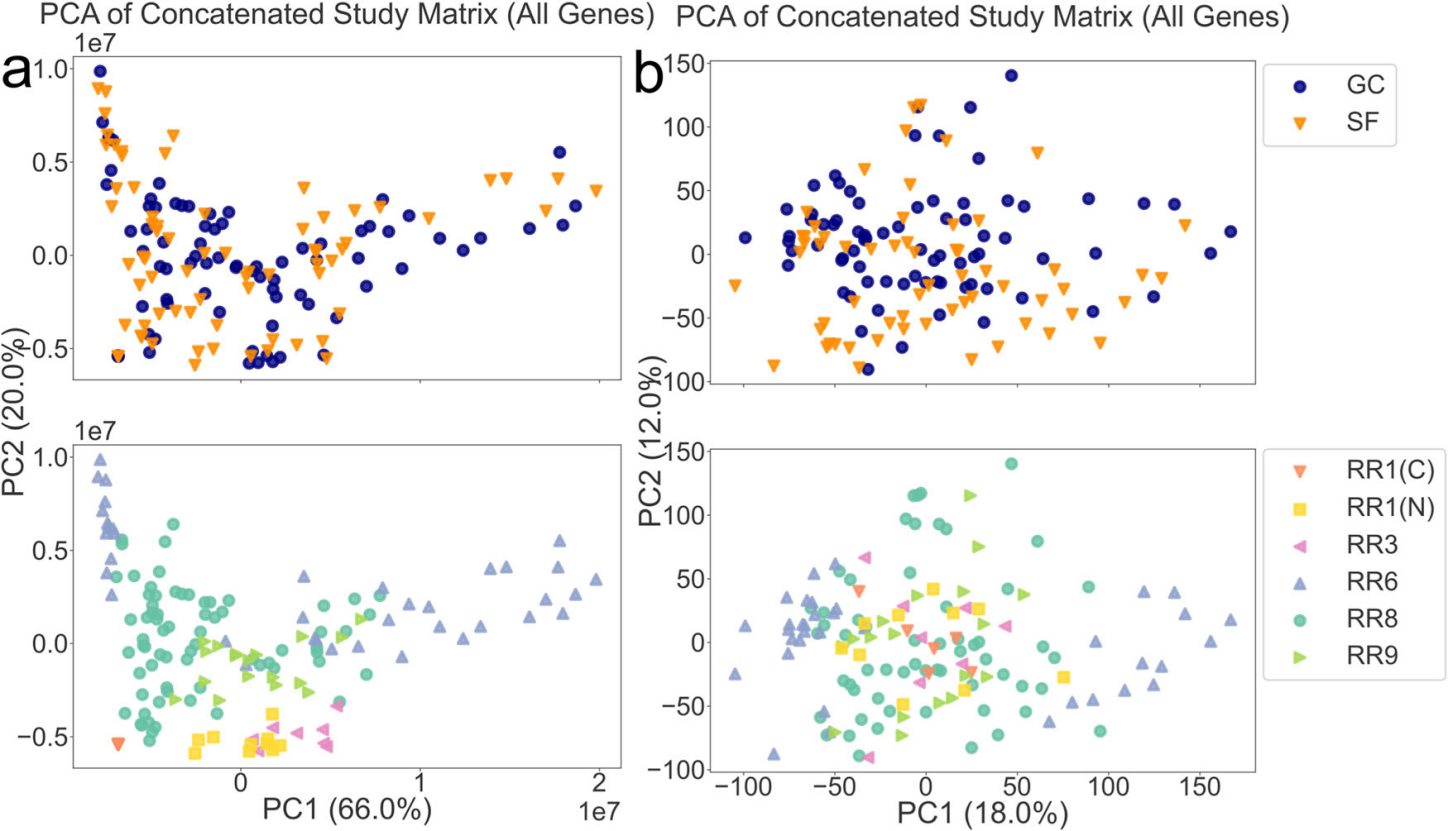
Dampening effects of systemic study differences through low gene-count prefiltering, log transformation, and Z-score standardization. **a** Mission-dominated clustering is present in raw and unnormalized counts data generated from RNA-seq of murine livers from Rodent Research missions 1, 3, 6, 8, and 9 based on top two principal components (PCs) of the concatenated datasets. Each point represents a murine liver sample from an RR mission, which has been labeled by spaceflown versus ground control status and mission-of-origin. The (N) and (C) distinguish between the NASA and CASIS RR-1 missions, respectively. **b** The *log* transformed and Z-score standardized datasets with low gene-count filtering show decreased mission clustering in top two PCs. PCA principal components analysis, SF Spaceflown, GC ground control, RR rodent research, CASIS Center for Advancement of Science in Space.

Principal component analysis (PCA) of unnormalized gene counts data concatenated across all datasets (Fig. 2a) shows 86% of total variability explained by PC1 and PC2. Clustering is dominated by RR missions instead of flight vs. ground status, indicating the strong presence of study-specific systemic effects. We found the signal-noise ratio (SNR) defined by the spaceflown signal to be low due to the noise introduced by study-specific systemic effects. A reduced gene set, filtering out pseudogenes and low count genes (17,733 genes, ~68% reduction), increased the total variability explained by PC1 and PC2 (92%), yet clustering was still dominated by RR missions. The systemic effects at the study-level were not removed with prefiltering at the sample and gene levels. The study-level systemic effects were reduced after the application of harmonization methods to gene counts that include the application of global *log*-transformations and within-study Z-score standardizations (Fig. 2c). The feature dimensionality at each preprocessing step is available in Supp. Table 1.

**Fig. 3:**
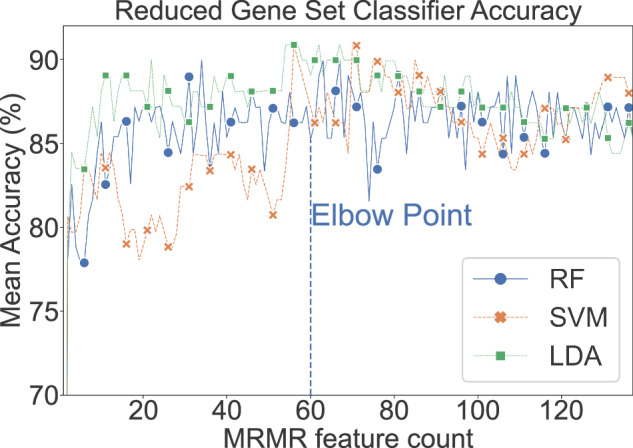
Selection of non-redundant features highly relevant to spaceflown classification with strong generalizability and minimal overfitting. Mean accuracy for RF, SVM, and LDA classifiers using a stratified 5-fold cross validation (CV) training scheme. The mRMR feature count reflects a completed iteration of the mRMR feature selection algorithm. The elbow point denotes the hyperparameter chosen for a reduced gene set that produces generalizable classification without overfitting to the training data. RF random forest, SVM support vector machine, LDA linear discriminant analysis, mRMR minimum redundancy maximum relevance.

We applied a systematic method for reducing dimensionality through the selection of a gene set that is representative of the most mutual information with minimal redundancy by applying the minimum redundancy maximum relevance (mRMR) criterion. From the training set, 5-fold cross validation was used to select an optimal mRMR feature set from the prefiltered, log-transformed, and standardized datasets. We applied three machine learning approaches to assess the ability to classify spaceflown vs ground control samples in our merged cohort. The mean classification accuracy (Fig. 3) of random forest (RF), support vector machine (SVM) and linear discriminant analysis (LDA) models at each iteration of mRMR show initially large increases in classification accuracy. As the feature selection method’s iterations approach the total number of available training samples (n = 108), overfitting is apparent per the steady plateauing in classification accuracy in all methods. In the training set, the iteration range between 55 and 80 shows a mean classification accuracy of 88% across methods. An elbow point at 60 iterations is used as the optimal number of mRMR-selected features where the accuracy values for all three classifiers start to plateau. The complete list of mRMR-selected features are available in Supp. Table 2. The increase in variance as the number of selected genes approaches the total number of samples is consistent with the bias-variance tradeoff commonly used to diagnose overfitting in models^21^.

**Fig. 4:**
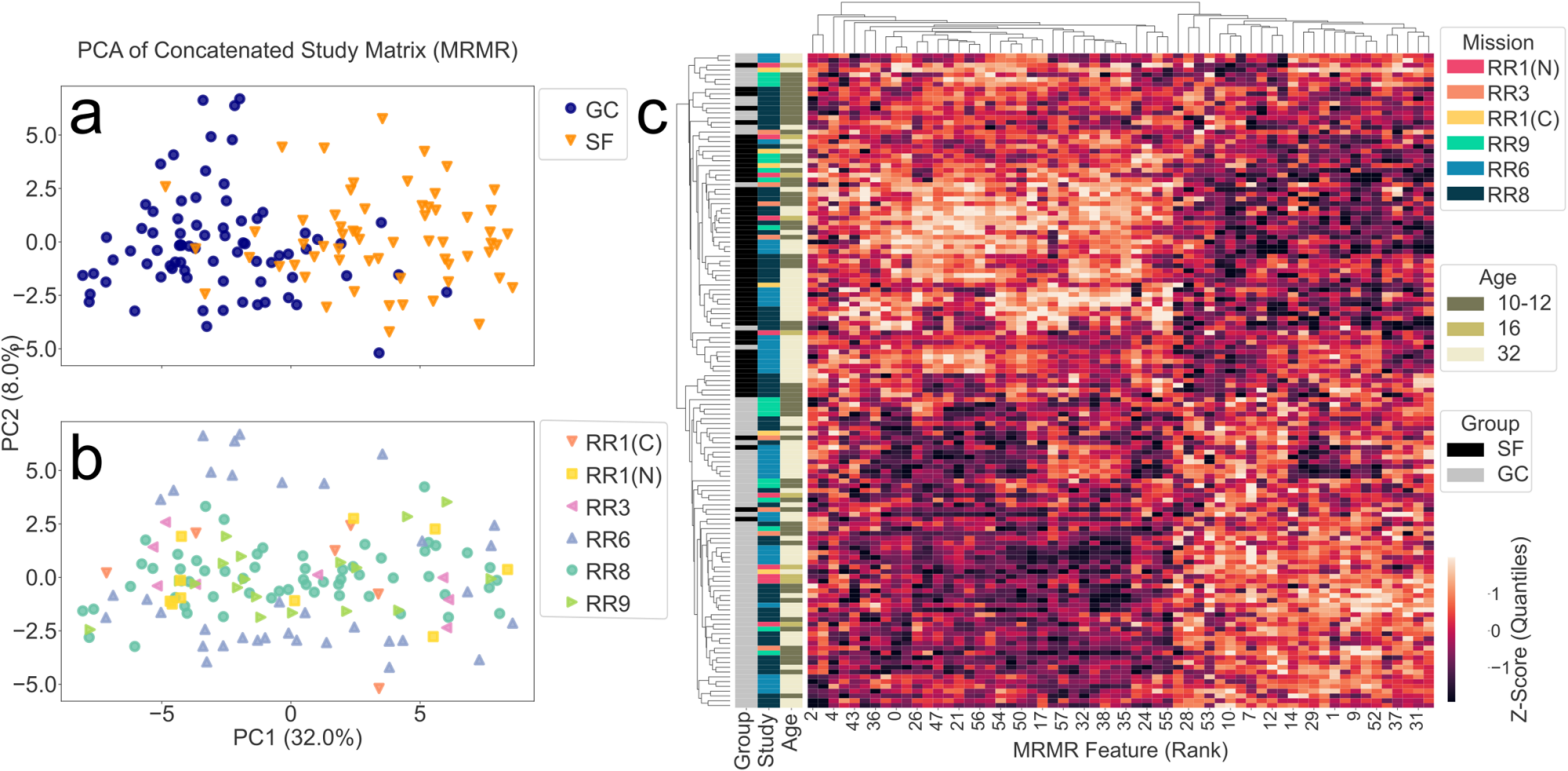
Spaceflown status is the primary driver of variability after mRMR feature selection. **a** Spaceflown-status dominates clustering in the *log* transformed, Z-score standardized, and mRMR subset datasets from RNA-seq of murine livers from Rodent Research missions 1, 3, 6, 8, and 9 based on top two principal components (PCs) of the concatenated datasets. Each point represents a murine liver sample from an RR mission, which has been labeled by spaceflown versus ground control status and mission-of-origin. The (N) and (C) distinguish between the NASA and CASIS RR-1 missions, respectively. Strong clustering is apparent along PC1 by spaceflown status. **b** The PCs encoded by RR mission. Overlapping clusters by mission are apparent along PC1 and PC2. **c** Clustermap of all samples using correlation based on Z-score quantiles ranging from [−1,1] for mRMR gene subset. Hierarchical clustering is completed on the sample-level and gene level. The left-hand side of the clustermap provides spaceflown, mission, and age labels for clustered samples. The sample clustering shows strong clustering by flight and ground status. Additionally, strong clustering by study is not present, which reflects a mitigation of systemic-study heterogeneity in the reduced mRMR feature subset. PCA principal components analysis; SF Space flown, GC ground control, RR rodent research, mRMR minimum redundancy maximum relevance, CASIS Center for Advancement of Science in Space.

The mRMR subset effectively changed the primary driver of variability to spaceflown status instead of systemic mission-level heterogeneities. Our preprocessing successfully increased the spaceflown SNR and dampened the noisy effects from study-specific systemic effects. The PCA and clustered heatmap using the 60-feature mRMR subset shows strong separation by spaceflown status (Fig. 4). PC1 accounts for 32% of the variance, and the plot encoded by treatment (Fig. 4a) indicates that spaceflown status is the strongest driver of separation along PC1. Mission is not the primary driver as a result of preprocessing steps (Fig. 4b), which enables model fitting to prioritize differences due to spaceflown status rather than between-study heterogeneities. This variability shift to spaceflown status is also reflected in the clustermap (Fig. 4c) with apparent clustering around flight vs. ground status and no apparent grouping by RR missions. Among the majority of samples clustering by spaceflown status, a deviation within the spaceflown cluster was attributed to age at launch. A subset of samples from RR8 and RR9 clustered independently of spaceflown status driven by similar ages at launch (10 weeks) for these missions. The older cohort of mice in RR8 (32 weeks) were not present within this cluster, however the older mice show independent clustering between RR1-CASIS, RR6, and RR8 missions. Although age-driven effects are present, spaceflown status is the primary driver of variability across datasets.

### Prediction performance

**Fig. 5:**
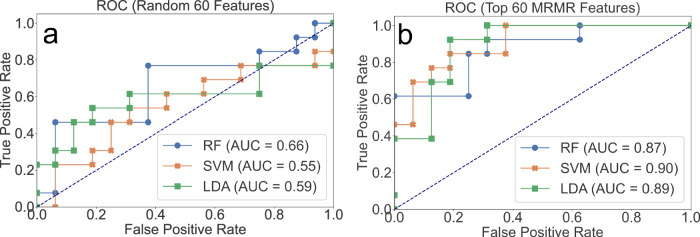
Prediction performance metrics for RF, LDA, and SVM classifiers significantly improve spaceflown sample classification. **a** Receiver operator characteristic curves for a random baseline of 60 features for RF, SVM, and LDA classifiers. The true positive rate and false positive rate for each classifier and their corresponding area under curve (AUC) metrics were calculated using the holdout testing set. The dashed line passing through the origin is included to show a poor binary classifier (AUC = 0.5). **b** ROC curves using the minimum redundancy maximum relevance (mRMR) selected subset shows significantly improved performance compared to the random baseline in discriminating spaceflight samples across RF (*p* value = 0.031), SVM (*p* value = 0.006), and LDA (*p* value = 0.01) models. Statistical tests were completed using Delong’s method on paired samples via the *pROC* R package. RF random forest, SVM support vector machine, LDA linear discriminant analysis, ROC receiver operator characteristic, mRMR minimum redundancy maximum relevance, AUC area under curve.

After training three classifiers to solve the classification problem of spaceflown versus ground control samples, they were evaluated on an independent test set. The performance of each classifier was measured on the test set using the area under the curve (AUC) of the receiver operator characteristic (ROC) curves and accuracy levels. For a baseline comparison, a random subset of the same number of features was sampled and used for model fitting according to the same training and testing split. The random sampling baseline repeated for 1000 iterations generated mean AUC values of 0.66, 0.55, and 0.59 for RF, SVM, and LDA, respectively (Fig. 5a). For the 60 mRMR-selected feature set, the AUC values were 0.87, 0.90, and 0.89 for RF, SVM, and LDA, respectively.

All classifiers performed at least 0.2 AUC higher relative to baseline estimates on an absolute AUC scale. SVM showed the largest absolute increase in AUC of 0.35. Therefore, using mRMR selected features, all classifiers performed significantly improved compared to their baseline in discriminating spaceflown samples across RF (*p* value = 0.031), SVM (*p* value = 0.006), and LDA (*p* value = 0.01) models. Statistical tests were completed using Delong’s two-sided method on paired samples between random and mRMR-selected features.

Scaling, prefiltering, and mRMR feature selection also improved average accuracies across additional methods tested based on invariant prediction methods. From the ensemble of methods included in the Causal Research and Inference Search Platform (CRISP)^22^, invariant risk minimization (IRM) and invariant causal prediction (ICP) show improved average test set accuracies relative to unnormalized data^23,24^. It is important to note that from the CRISP ensemble, we only selected modules that were robust and applicable to classification problems so that we could have a direct comparison with other methods tested in this study. In the testing set, accuracies in classification of spaceflown mice across all methods were found to be 0.72, 0.79 and 0.79 for RF, SV and LDA and 0.52, 0.78 and 0.67 for non-linear IRM, linear ICP and linear IRM, respectively (Supp. Fig. 1).

### Feature importance between methods and interpretability

**Fig. 6:**
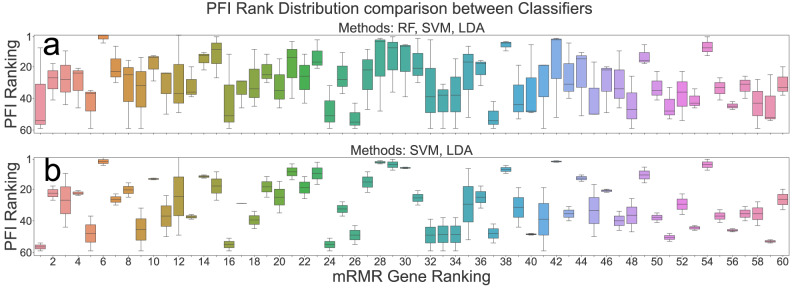
The standardized PFI-based gene ranking shows concordance across methods. **a** Box and whisker plot of permutation feature importance rankings from trained random forest, linear discriminant analysis, and support vector machine classifiers against the ordered mRMR feature subset. **b** Removing RF to show strong concordance between LDA and SVM gene rankings. PFI permutation feature importance, SVM support vector machine, LDA linear discriminant analysis, RF Random Forest, mRMR minimum redundancy maximum relevance.

Permutation feature importance (PFI) ranking of top mRMR selected features was computed to determine the relative importance of features between SVM, LDA, and RF classifiers. The trained classifiers do not rank genes in the same order as mRMR (Fig. 6a). The boxplot ranks importance in ascending order and shows concordance between classifiers based on the tightness of the interquartile range. For instance, the genes *Asb13*, *Zbtb21*, *Rexo2*, *AA645442*, and *Bag2* are the top five concordant genes across all methods and the mRMR ranking. Excluding mRMR, *AA645442*, *Asb13*, *Tlr11*, *Zbtb21*, and *Tgfbi* are the top five concordant genes across classifier rankings. Including RF increased the interquartile range, and we found strong concordance between SVM and LDA rankings based upon >50% overlap in interquartile ranges <5 (Fig. 6b). For this subset of gene rankings from SVM and LDA, the top five concordant genes are *Rtp3*, *Cxcr3*, *B3gnt9*, *Tubb4b*, and *Matn2*.

The concordant genes were compared against genes known to be associated with liver and space studies, specifically those related to *Ppara* (*Nr1c1*, *Nr1c2*, and *Nr1c3*), cytochrome *P450* (*Cyp1a1*, *Cyp1a2*, *Cyp2c23*, *Cyp2e1*), oxidative stress (*Aqr*, *Cygb*, *Fmo2, Gpx6, Mb, Nox1, Tmod1, Ucp3, Idh1, Nox4, Scd1*, and *Serpinb1b*), and apoptosis (*Cdkn1a*)^9,25–27^. Due to the heavily reduced nature of the mRMR gene set, each selected feature is a representative of a group of highly correlated features. For this comparison, a set of highly correlated genes were mapped to each gene from the concordant mRMR genes. The threshold for each mRMR expanded gene set was determined using the correlation distance between the mRMR selected feature *Txnip* and its known regulator *Ppara*, which is involved in regulating liver processes^28^. *Ppara* was identified within the nearest 250 correlated genes (98^th^ percentile) to *Txnip*. This nearest correlation distance was used to produce an expanded set of 250 genes from each mRMR gene. In addition to *Txnip*, we identified three other mRMR-selected genes (*Asb13, Bag2*, and *Zbtb21*) also correlated with *Ppara* genes *Nr1c1* and *Nr1c2*. The *Zbtb21* cluster contains gene *Cdkn1a*, which encodes a potent cyclin-dependent kinase inhibitor with a regulatory role in S-phase DNA replication, DNA damage repair, and apoptosis^26^. Two additional mRMR-selected genes (*Rtp3* and *AA645442*) were highly correlated with gene *Aqr*, which was upregulated in a previous study showing oxidative stress from gene expression analysis of murine livers exposed to spaceflight from STS-118^27^. Two mRMR gene clusters (*Bag2* and *Rexo2*) included gene *Idh1*, which was downregulated and related to oxidative stress from the previous STS-118 study. No overlaps between cytochrome *P450* were identified within the concordant gene clusters.

### Overlapping gene set enrichment analysis

We performed gene set enrichment analysis (GSEA) to investigate genes contributing to gene ontology biological processes (GO BPs) that were significantly enriched (adjusted *p* value ≤ 0.1) in either the SVM merged-study analysis or single-study differential gene expression (DGE) analyses, focusing on studies with most number of samples. Here, we focused on SVM for merged-study analysis because we found this method most robust for GSEA and gene-level interpretation. In the SVM merged-study GSEA we identified 276 significant GO BPs, and in the DGE single-study GSEA of RR1 (NASA), RR6, RR8, and RR9 we identified a total of 263, 187, 319, and 238 significant GO BPs, respectively. The overlap of significant BPs identified in single-study analyses with the SVM merged-study analysis ranged between 13% and 41% (Table 2). Many of the single-study BPs were not shared across missions, highlighting study and strain differences. Genes contributing to core enrichment of these BPs highlighted major histocompatibility complex (*Mhc*) class I haplotypes (*H2* region genes) and strain genetic background mutant genes. In addition, we identified a set of unique BPs from SVM merged-study analysis that were not detected or significant in single-study analyses. For the set of GO BPs identified within the SVM merged-only set (Supp. Table 3), genes contributing to core enrichment (Supp. Table 4) included those broadly related to immune response (*Xcl1*), inflammatory response (*Il1b*, *Ccl5*), and liver metabolic processes (*Ccr2*, *Egfr*, *Il1rn*, *Xbp1*).

**Table 2:** Overlapping Significant Gene Ontology (GO) Biological Processes (BPs) between merged-study and single-study analyses

Number of Significant GO BPs by RR Mission^a^ and Group^b^	RR1 (NASA)	RR6	RR8	RR9
Total in Single-Study	263	187	319	238
Single Study Only^**c**^	227 (86.3%)	137 (73.3%)	198 (62.1%)	139 (58.4%)
Single-Study and Merged-Study^**d**^	36 (13.7%)	50 (26.7%)	121 (37.9%)	99 (41.5%)
Overlap with Merged-Study Total (*n* = 276)^**e**^	164	253	257	241
Merged-Study Only	128	203	136	142

^a^Number of Gene Ontology (GO) biological processes (BPs) returned from GSEA using ranked fold changes for individual RR datasets and ranked SVM-coefficients using the merged datasets.

^b^The significant GO BPs are determined based upon an adjusted *p* value cutoff of 0.1.

^c^The subset of the GO BPs that were significant only in single-study GSEA and its percentage of the total.

^d^The subset of the GO BPs that were significant in both single-study and merged-study GSEAs and its percentage of the total.

^e^The number of GO BPs from the merged-study GSEA that were also picked up from single-study GSEA.

GO BPs from GSEA of individual RR missions and SVM merged-study analysis (Supp. Fig. 2) show overlapping BPs between all missions except for RR1 (CASIS) (*n* = 6) and RR3 (*n* = 8), which did not return any significant BPs. The distribution of normalized enrichment scores for GO BPs identified at the single-study level compared against the SVM merged-study level broadly shows that most BPs with significance had consistent directionality in single study and merged analysis (Supp. Fig. 3). From the 22 unique sets with at least one overlap, eight instances included the GO BPs identified from the SVM-based analysis. The overlapping GO BPs may indicate a shared biological response in RNA expression from spaceflown cohorts across multiple rodent research missions. Clustering analysis of GO BPs overlapping at least three RR missions and SVM included parent terms related to *adaptive immune response*, *mitochondrial translation*, *cell killing*, and *regulation of defense response* (Supp. Table 5).

## Discussion

In this study, we demonstrated the use of ML methods on harmonized RNA-seq data from six studies to find robust and generalizable features that discriminate between spaceflown vs. ground control livers. Our study relies on short-duration RR missions to the ISS which are each small sample size and not amenable to ML methods. While our harmonized analysis has a sample size larger than most single spaceflight studies, the total study size is still not sufficient for complex machine-learning algorithms. Using low-parameter configurations of models and dimensionality reduction to minimize overfitting, we enabled the training of classification models that showed strong predictive capabilities for spaceflown status. Pipeline consistency, log transformation, and Z-score parameter estimations applied at the cohort-level, prior to merging, shifted the primary signal to spaceflown status from noisy mission-level features. This approach is in-line with batch normalization practices in ML-model training^29^ and consistent with other work that have shown RNA–seq expression measurements cluster by tissue rather than laboratory of origin given standardized preprocessing transformations^19,30^.

Models trained on effectively harmonized data fit better to the shared signal across all studies rather than any single dataset^20^. Our merged analysis has several advantages over single-study analyses. First, we retained pathways signals found in single studies and previously reported spaceflight studies including *Ppara*, lipid dysregulation, apoptosis (*Cdk1a*), oxidative stress (*Aqr* and *Idh1*), and processes previously found dysregulated by spaceflight^9,13,26–28^. Second, we found increased generalizability across studies and more power to detect significant pathways by identifying enriched BPs that were significant in the merged-study-only group but were not detected or not significant in the single-study analyses. These pathways were found to be broadly related to immune response (*Xcl1*), inflammatory response (*Il1b*, *Ccl5*), and liver metabolic processes (*Ccr2*, *Egfr*, *Il1rn*, *Xbp1*). Thirdly, for pathways that did not reach significance in the merged-study analysis, but were significant in single-studies, we found strain-specific effects such as *H2* genes, which are related to the major histocompatibility complex (*MHC*) genomic region known to vary in expression between haplotypes and inbred strains^31^. These highlight the strength of our approach.

For heterogeneous, multistudy datasets, we found that it was key to balance key study characteristics between training and testing subsets. Despite balancing, the sex of the study samples was majority female and therefore no generalizable conclusion can be made for sex differences in this analysis. To further trust in the robustness of our testing results, we approximate data transformations and perform feature selection excluding the testing set to ensure fairness during model evaluation. The sequential nature of the mRMR framework during model training was effective in showing the onset of overfitting and has been used as a robust method to avoid overfitting of SVM and RF models by others performing analysis of RNA-seq data^30^. Hyperparameter optimization was not explored in this analysis due to lack of available data for validation, but methods such as nested k-CV can be utilized to fine-tune models once new data are available.

For spaceflight studies, meaningful information and interpretability of ML-based analyses and results are critical to maximize limited resources and scientific return. Classification performance alone is insufficient for model interpretation. ML methods that can provide gene-level coefficient estimates are more accessible for interpretation, namely SVM with a linear kernel and LDA. RF notably lacks feature coefficients, but mutual information-based measures can be used to calculate feature importances. Similarly, we found that modules available from the CRISP ensemble were not suitable to calculate feature importance due to lack of accessibility to the feature space from the ensemble^22^. Therefore, we focused on ML-methods that we could rely on robustness and interpretability abilities. We used PFI to standardize gene rankings to generate gene-level metrics allowing us to compare feature importance across all predictive ML-models. When comparing the rankings derived from the mRMR (per F-statistic and Pearson correlation) against the PFI-ranked features within each method, there are rank differences between ML classifiers and mRMR. Ensemble methods like RF rank features differently than SVM and LDA although all models have similar AUC values. PFI-derived gene importances enable these between-model comparisons which are not available in aggregate-level only performance metrics.

Our approach of combining and harmonizing multiple data sets provided sufficient data to train predictive models and consistently identify important genes. The minimally redundant gene sets are beneficial for prediction approaches, but do add limitations to understanding the relevant pathways. Expansion of the consistently identified reduced gene sets into larger representative sets of genes included genes (*PPARα*, *Cdkn1a, Aqr, Idh1*) previously found dysregulated by spaceflight in murine liver^9,13,26,27^. Our study relies on short-duration rodent research missions to the ISS which limits the time to observe the development of relevant disease pathology. Therefore, the observed altered genes are assumed to be correlated to diseases in the organ of interest (e.g., liver). As more spaceflight data is generated, we can extend this analysis and identify robust signatures consistently dysregulated across spaceflight missions.

An interpretation limitation of the mRMR reduced subset for GO over-representation analysis is that highly correlated sets of genes are filtered out since each mRMR selected gene represents a set of correlated genes. The lack of correlated sets of genes results in low numbers of biological processes from GO annotations. For methods like GSEA, which require a ranked metric on all genes, large feature reduction is incompatible, however, we addressed this issue by expanding the selected features with correlated sets. When all genes are included for fitting compatible models, like SVM, the returned GO BPs did show considerable overlap with those identified from the classical DGE analysis at the study level. SVM is known to work with small datasets with high dimensionality^32^, and its coefficient estimates include directionality. However, the standardized gene scoring using PFI does not include directionality, therefore gene upregulation versus downregulation cannot be determined from this metric alone and need to be coupled with experimental designs for further validation.

This study developed a robust way of next-generation sequencing data harmonization for individually low-count experimental samples such that more generalizable machine-learning methods can be utilized to identify genes altered in combined studies of spaceflown murine livers. While specific biological pathways were successfully identified as involved in the murine response to spaceflight, larger follow-up studies are needed to make any concrete conclusions about the biological findings and their association to liver disease. Therefore, future studies are required for external validation of the pipeline developed in this study and the identified machine learning-based gene scores.

## Methods

### Selected cohorts

All study cohorts are selected from the NASA rodent research (RR) missions conducted on the ISS^33^. Open Science Data Repository (OSDR) datasets from five NASA Rodent Research (RR) missions with murine liver RNA-Seq data are included in this analysis (RR-1 NASA^34^ & CASIS^35^, RR-3^36^, RR-6^37^, RR-8^38^, and RR-9^39^). Each rodent cohort has steadily iterated upon hardware configurations to support rodent experiments in space and these missions will continue to study the physiological effects of spaceflight in rodents to inform countermeasure development. From these missions, transcriptomics data generated for murine liver samples have been maintained on NASA OSDR^40^. Each mission included in this study has spaceflown (via the ISS) and ground control cohorts of mice. The spaceflown and ground control pairs within each study controlled for factors including age, sex, strain, and housing. Detailed information on the experimental setup within each study is available on the NASA OSDR platform^5^. Between studies, experimental conditions differed as noted in metadata Table 1. RNA-seq counts data across all studies were represented as an NxD matrix with individual samples and ENSEMBL gene identifiers along the rows (*N*) and columns (*D*), respectively. A total of 153 spaceflown and ground control samples were available across all studies, and 16 were removed as outliers per the Outlier Removal process described below. An additional two spaceflown samples from RR-1 were removed due to known quality issues associated with live animal return (LAR) to Earth prior to euthanasia, which confounded the space flight response due to the stress of LAR. Samples from RR-1 NASA and RR-3 were reprocessed in GLDS-168 to test the effectiveness of External RNA Control Consortium (ERCC) spike-ins. The technical replications of RNA-Seq data generated in GLDS-168 were removed from analysis to maintain a balanced representation of samples across all studies^41^. All measurements were taken from distinct sample tissues.

### Dimensionality reduction

The ENSEMBL gene identifiers were initially subset to those available across all experiments. As all cohorts were processed through NASA OSDR, gene transcripts were consistent across all count matrices. Pseudogenes were annotated using the R package *biomaRt* and the *mm10* reference assembly, and were excluded from the analysis^42^. After pseudogene removal, the total counts distribution across all experiments still reflected a sparse data set with a large proportion of genes associated with a low number of counts. Gene-level counts that are very low (<5) are not informative for training and would normally be filtered out in DGE analysis^43^. Using a minimum count threshold of 5, a distribution of gene-sample counts was generated to identify a feasible cutoff for sample coverage (Supp. Fig. 4). The tails of the distribution (Supp. Fig. 4a) shows that ~34% of genes are less than five across all samples (sample coverage = 0%) and ~32% of genes are greater than five across all samples (sample coverage = 100%). The tail-removed distribution (Supp. Fig. 4b) indicates that 50% of the remaining genes represent a sample coverage of only 4%. Such low sample coverage means that the data are still sparse after removing zero-sample coverage genes. Thus, a conservative threshold based on the mean sample coverage (17%) was used to remove genes that were not well represented in samples across datasets.

### Principal components analysis

The data processing pipeline applied to RNA-seq datasets included multiple steps. At each pipeline step, PCA was applied (Fig. 2). The first two PCs can indicate primary drivers for variability in the harmonized data. Clustering of samples along PC1 and PC2 were used to diagnose pipeline effectiveness. A pipeline step is considered effective if it addresses an aspect of experimental heterogeneity. Each pipeline step and its purpose in mitigating a source of heterogeneity are detailed in the sections below.

### Outlier removal

Although sample size is notably minimal across studies, poor quality samples must be pruned prior to model fitting. If the sample was not within its respective mission clusters from PC1 and PC2 of unnormalized data, it was excluded from analysis. RNA integrity number (RIN) was reviewed across all RNA-seq tissue samples to estimate the integrity of total RNA and the presence of degradation products. Absolute RIN cutoffs were considered but not deemed robust enough to apply across all experiments^44^. Rather, if any sample’s RIN value deviated below the mean for all spaceflight and ground control samples (*μ*_RIN_ = 8.02) it was compared to others from the same experiment.

### Addressing data heterogeneity

It is important to note, there are multiple factors that affect data heterogeneity in this study, such as systematic study-specific effects (e.g., batch effects, RNA-seq library preparation and sequencing protocol), differences in mice strains, average age and gender^45^. These factors are present in the data pre-transformation based upon strong clustering by RR mission (Fig. 2a). Therefore, the following steps were taken to address data heterogeneity.

### Normalization of RNA-seq counts

Normalization of RNA-seq typically addresses varying sequencing depths, gene lengths, and RNA compositions. Here, RNA-seq counts data were transformed to account for sequencing depth and RNA composition. Both factors are corrected using *DESeq2*’s median of ratios (MoR) to ensure a fair comparison of gene expression between samples within any given study. Other methods evaluated include log counts per million, transcripts per kilobase million, reads per kilobase of exon per million^46^. MoR was preferred because of its sample-specific size factor for normalization and synergy with differential expression analysis^47^. MoR is applied to all RNA-seq data prior to partitioning into training and testing data sets.

### Normality transformations

Raw RNA-seq count data are not normally distributed, sparse, and heteroscedastic. Sparsity was partially addressed by prefiltering low sample coverage genes, but the remaining genes showed heteroscedastic trends. Genes abundant in the liver were expressed with a high mean and high variance. This gene-count bias produces extreme outliers that will negatively impact ML-model cost functions and optimizations that rely upon regression-based methods. If gene-count bias is not corrected, regression-based methods do not treat covariates independently and complicate gene comparisons in downstream analysis. To address gene count bias, *log* transformation was applied to the data. The log transformation is known to reduce the effects of gene outliers in RNA-seq data and produce a more normal distribution^47^. Alternatives to log transformation include regularized log, variance stabilizing transformation, and trimmed mean of M values^47^. For gene counts *x*, the *log*_2_(x + 1) transformation was sufficient in reducing the mean and variance of genes abundant in the liver. After log transformation, Z-score standardization at the individual gene level was applied to further normalize the data. Standard scaling gene counts across all samples transformed gene counts *x* into standardized gene scores *Z*, where $Z=\frac{x-\mu }{\sigma }\,$, $\mu$ is the mean gene count across all samples and $\sigma$ is the respective unit variance. This method of scaling adjusts global properties of measurements for individual samples so they can be more appropriately compared^45^. This step is crucial to ensure fair comparisons across studies and reduce the effects of system study effects. Alternatives to this standardization were considered using batch effect estimation and removal via linear or empirical Bayes methods via the *limma* and *ComBat-seq* R packages^48–50^. Z-score is preferred for comparisons and model training because it maps the dynamic range for counts data into a standardized metric based upon global data properties^51^. PCA of the transformed data (Fig. 2c) proved effective in reducing the RR mission clustering previously noted in the data.

All data preprocessing steps were completed study-by-study prior to concatenation into a single matrix to allow for across-data comparisons. After concatenation, the pooled counts matrix are mapped to a target vector, *c*, where each element in *c* corresponds to a spaceflown or ground sample. The target vector is one-hot encoded to set up the binary classification problem for feature selection and model training.

### Minimal redundancy maximum relevance (mRMR) feature selection

Once the data was harmonized, the minimum redundancy maximum relevance (mRMR) criterion was applied to select top informative features from the training data set. The mRMR criterion is a feature selection technique developed according to maximal statistical dependency. It is based upon mutual information via a combination of max-relevance and min-redundancy and a two-stage feature selection^52^. The functions used for relevance and redundancy are F-statistic and Pearson’s correlation, respectively. These functions map the numerical input data (standardized gene scores) to the target vector (spaceflight encoding).

The sequential algorithm iterates through the entire gene feature set to compute each gene’s relevance with respect to spaceflight across all samples. Concurrently, the redundancy for each gene is computed with respect to selected features. A gene is added such that the relevance is maximized and redundancy is minimized. Here, the incremental search and selection process was stopped once the number of selected features was equal to the total number of available samples. All binary classifiers were trained using the mRMR feature subset at each iteration.

### Cross-validation training and data partitioning

Due to the heterogeneity in experiments, training and testing sets are balanced across the experimental factor differences as noted in Table 1. The distribution of factor levels maintained a sex, age, and strain-balanced training and testing cohort using an 80-20 training and independent testing split (Supp. Fig. 5).

The training set was used for estimating Z-score transformation parameters and mRMR hyperparameter tuning. Data partitioning during hyperparameter tuning was completed according to a 5-fold cross validation. Each fold was stratified to preserve the relative percentage of spaceflight and ground controls.

### Classification machine learning models

The supervised machine learning models trained in this analysis include random forest (RF), support vector machine (SVM), and linear discriminant analysis (LDA). RF is an ensemble learning algorithm that constructs randomized decision trees. The predictions from these trees are aggregated by averaging to predict class membership probabilities^53^. The *RandomForestClassifier* implementation from the *scikit-learn* (v1.0.1) Python package was used. The RF hyperparameters for number of estimators (*100*), max depth (*5*), and minimum samples per split (*2*) were overridden from default configurations. During RF fitting, bootstrapping, balanced class weighting, and out-of-bag scoring were enabled. SVM in its supervised classification configuration finds a hyperplane that best separates classes in a feature space. SVM uses kernels to transform the data from input to feature space for classification^54^. The *LinearSVC* implementation from *scikit-learn* was used with squared hinge loss, L2 penalization, and regularization (*c* = 1.0). LDA is a method that maximizes the ratio of between-class variance to the within-class variance to guarantee maximum separability. The main objective of this class-dependent LDA is to maximize the described ratio enough to adequately separate classes^55^. The *LinearDiscriminantAnalysis* implementation from *scikit-learn* was used with overridden defaults for the solver *(eigen*) and shrinkage (*Ledoit-Wolf lemma*) parameters. Model performance was evaluated using the area-under-the-curve (AUC) from the receiver operator characteristic (ROC) curve generated from the testing set. For a baseline comparison, a random subset of the same number of features was sampled and used for model fitting according to the same training and testing split. The random sampling baseline repeated for 1000 iterations. The *pROC*(v1.18.0) R-package was used to test the significance of incremental value in AUC values from mRMR selected features as compared to the randomly selected baseline features^56^. From the partial ROC package, the paired test using DeLong’s two-sided method was applied to the same set of samples for the baseline random feature subset and the mRMR subset.

Invariant prediction methods included in the Causal Research and Inference Search Platform (CRISP) ensemble were also explored^22^. From the full set of algorithms available in the CRISP ensemble^22^, the invariant prediction methods for invariant risk minimization (IRM) and invariant causal prediction (ICP) were tested. IRM is a method which estimates nonlinear, invariant, causal predictors from multiple training environments to enable out-of-distribution generalization^23^. ICP searches for a combination of features that can correctly predict a target variable across multiple training environments^24^.

### Permutation feature importance

PFI was applied to trained classification models to generate standardized gene scores from the holdout testing set. The *permutation_importance* implementation from *scikit-learn* was used based upon overridden default configurations for a number of random permutations (100) and scoring (*AUC*). The PFI heuristic was evaluated on the holdout testing set with a response vector encoded by spaceflown vs. ground status. PFI provides ML-based methods with a standardized metric for comparisons. The ordered standard deviation of mRMR gene rankings for classifiers was used to determine concordance. PFI applied to multicollinear features may return low importance scores for all features. Highly correlated genes in the data were addressed using the mRMR method for selecting non-redundant features through Pearson correlation and F-statistics. The optimal point for PFI comparisons in this analysis only included the subset of minimally correlated genes. The nonnegative scores generated by PFI do not reflect the directionality between the input gene features and the response vector.

### Differential gene expression and gene set enrichment analysis

RNA-seq counts and metadata for each study were obtained from NASA OSDR (Table 1). The pipeline used to generate these data has been previously published^5^. A total of 16 samples were determined as outliers according to the criteria described earlier. Technical replicates were removed from GLDS-168, which contained samples processed with and without ERCC spike-in controls. DGE analysis on RNA-seq data was performed using the *DESeq2* (v 1.30.1) package. RNA-seq counts were normalized using the median-of-ratios method prior to DGE analysis between the spaceflown vs. ground control experimental groups. For individual RR missions, the calculated log_2_(fold-change) values were used to rank genes for enrichment analysis using the *ClusterProfiler* (v3.18.1) R-package. For the merged analysis, the coefficients from the trained SVM estimator were used to rank genes for enrichment analysis. A Benjamini-Hochberg (BH) adjusted *p*-value cutoff of 0.1 was used to determine significantly enriched BPs. A BH-adjusted *p* value cutoff of 0.9 was used to form the set of GO BPs for comparison between merged and single-study analyses. Reduced lists of GO processes were determined using the *rrvgo*(v3.16) R-package. Top contributing genes GO BPs were determined by aggregating the subset of genes that contribute most to the enrichment results and filtering out genes present in less than ten significantly enriched GO BPs.

### Reporting summary

Further information on research design is available in the Nature Research Reporting Summary linked to this article.

### Supplementary information

Supplementary Information

Reporting Summary

## Data Availability

The datasets analyzed in this study are publicly available RNA-seq counts data and metadata on NASA’s OSDR under the OSDR-47, -168, -242, -245, and -379 database entries. The harmonized datasets generated in this study can be replicated from the code available on NASA’s Github account under the *trrac* repository at https://github.com/nasa/trrac.

## Code availability

The underlying code, including Jupyter notebooks and scripts, for this study, is available on NASA’s official Github account under the *trrac* repository at https://github.com/nasa/trrac.
