# Supplementary material for: Harmonizing heterogeneous transcriptomics datasets for machine learning-based analysis to identify spaceflown murine liver-specific changes
Source: NPJ Microgravity. 2024 Jun 11;10:61. doi: 10.1038/s41526-024-00379-3 (PMC11167036; doi:10.1038/s41526-024-00379-3)
Supplement: Supplementary file 1 — Supplementary Information [file 41526_2024_379_MOESM1_ESM.pdf]

Harmonizing Heterogeneous Transcriptomics Datasets for Machine Learning based Analysis to Identify Spaceflown Murine Liver-specific changes

Hari Ilangovan[1*], Prachi Kothiyal[2], Katherine A. Hoadley[3], Robin Elgart[4], Greg Eley[2], Parastou Eslami[5]

[1]Science Applications International Corporation (SAIC), Reston, VA 20190, USA

[2]Scimentis LLC, Statham, GA 30666, USA

[3] Department of Genetics, Computational Medicine Program, Lineberger Comprehensive Cancer Center, University of North Carolina at Chapel Hill, Chapel Hill, NC 27599, USA

[4]University of Houston, Houston, TX 77204, USA

[5]Universal Artificial Intelligence Inc, Boston, MA 02130, USA

[*]ilangovan.hari@gmail.com

Supplementary Figures

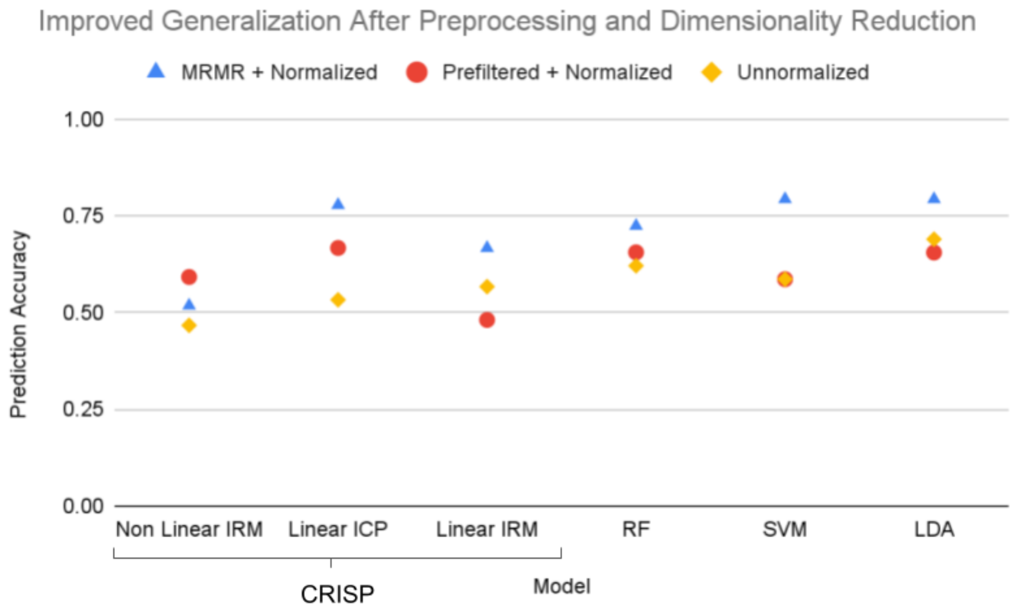

Supplementary Figure 1: Improved performance using preprocessed data for SVM, RF, LDA and invariant methods from CRISP The mRMR subset and standardized data show better performance on the testing set than unnormalized data across all methods. The mRMR and normalized dataset showed the best performance for RF, SVM, LDA, linear ICP, and linear IRM. IRM: invariant risk minimization; ICP: invariant causal prediction ; CRISP, causal reasoning and inference search platform; RF: random forest, SVM: support vector machine; LDA: linear discriminant analysis

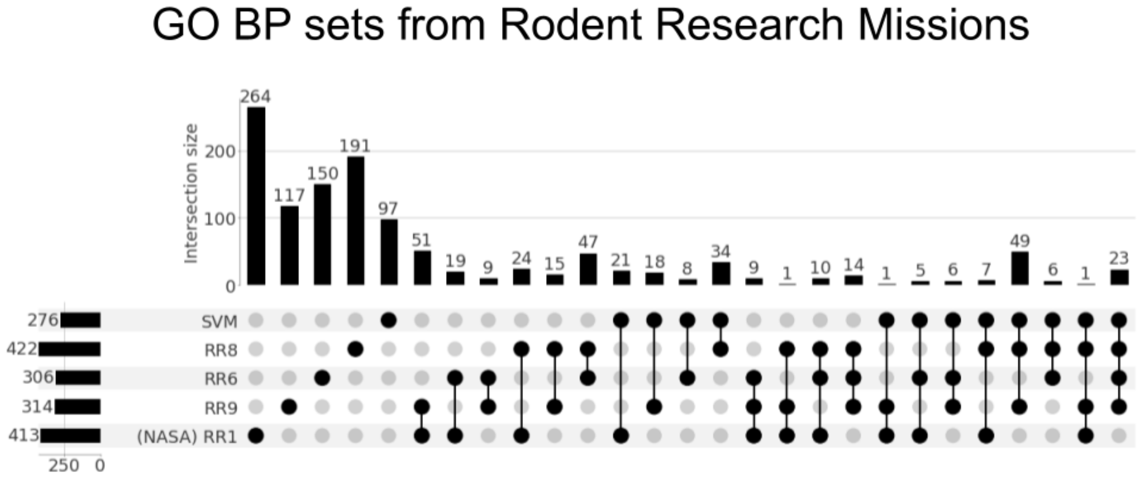

Supplementary Fig. 2: Fold change-based ranking metric at single-study level and SVM-based ranking at merged-study level show strong overlapping GO BPs

Sets included in the UpSet plot shows the Gene Ontology biological processes returned from *DESeq2* analysis at an individual study level versus SVM analysis from the merged-study harmonized analysis. The rows indicate rodent mission titles for the study-level analysis and *SVM* for the combined study analysis. The columns indicate intersection sets that are based upon the filled-in dots.

RR, rodent research; SVM, support vector machine; GO gene ontology; BP biological process;

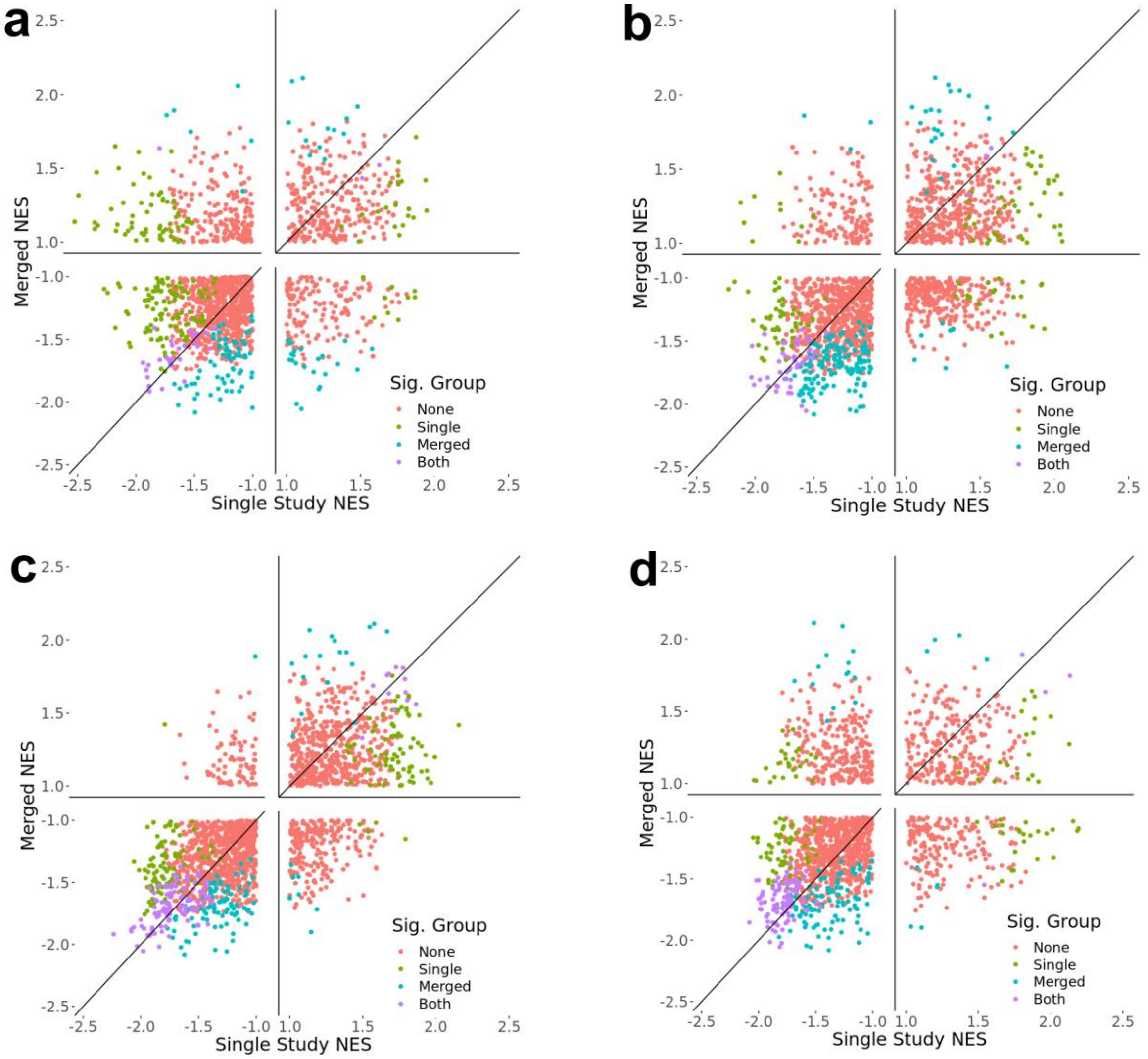

Supplementary Fig. 3: Normalized enrichment score from GSEA compares between single mission analysis versus merged SVM analysis.

a: The gene ontology biological processes (GO BPs) returned from GSEA for single-study analysis and the SVM merged study analysis with each point on the normalized enrichment score (NES) scatter plot representing a GO BP returned from both the single-study and SVM merged study analysis. Each GO BP is labeled based on its significance (p-value ≤ 0.1) in both single study and merged analysis, significance in the SVM merged analysis only, significance in the single study analysis only, and not significant (p-value > 0.1) in either analysis for RR1 (NASA) **b:** RR6 **c:** RR8, **d:** RR9. NES, Normalized Enrichment Score; GO, Gene Ontology; BP, Biological Process; GSEA, Gene Set Enrichment Analysis; SVM, support vector machine

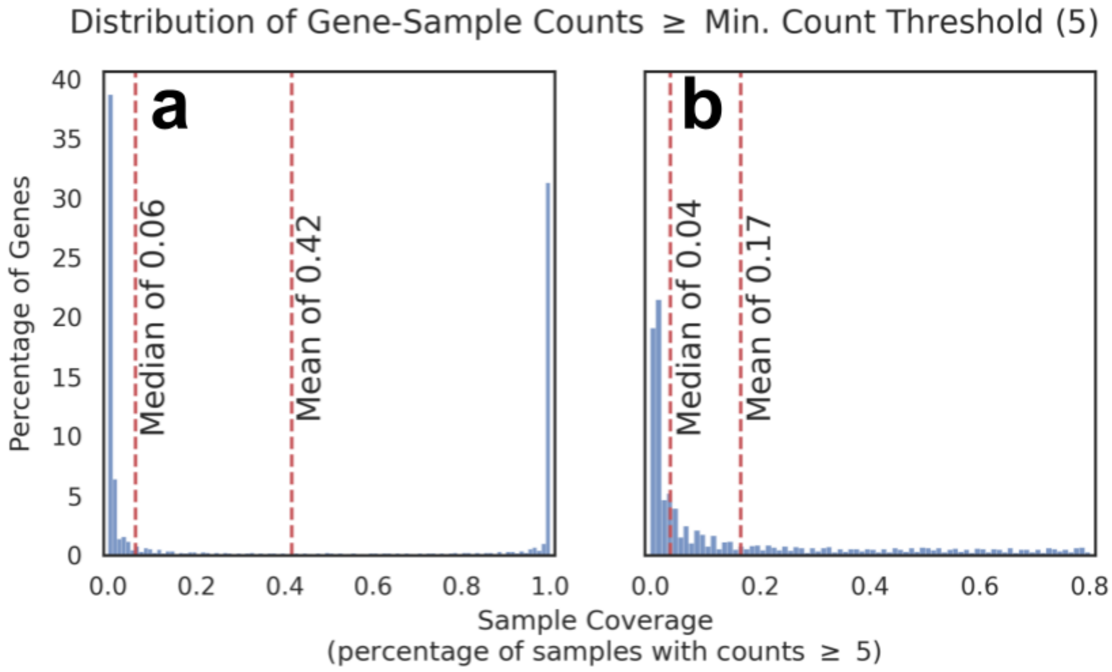

Supplementary Fig. 4: **Histogram of genes with counts exceeding the minimum count threshold across all samples a:** Histogram of the gene count measurements exceeding the minimum count threshold of 5 across all samples. The y-axis corresponds to the percentage of genes that belong to the percentile of sample coverage denoted by the x-axis (bin size = 1%). The observations corresponding to no coverage (sample coverage = 0%) and high coverage (sample coverage ≥ 80%) are referred to as tails. The distribution with tails and **b:** without tails are included with median and mean calculations overlaid. The median indicates sample coverage percentage associated with the 50th percentile of genes. The mean indicates the average sample coverage across all gene count measurements.

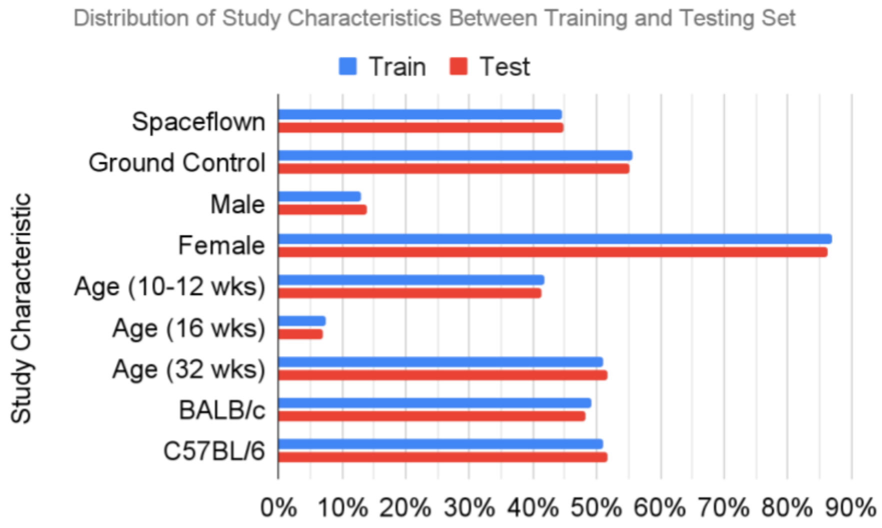

Supplementary Fig. 5: The percent composition of study characteristics in the training and testing set for spaceflown status, sex, age, and strain. The spaceflown status, sex, age at launch, and strain for NASA Rodent Research missions 1, 3, 6 ,8, and 9 for n=13. An 80% training and 20% testing split is used to determine the relative composition between study characteristics, which are within 5% across each characteristic. The age refers to the age at launch in weeks.

Supplementary Tables

Supplementary Table 1: Feature counts at each dimensionality reduction step

Step	Description of Preprocessing	Number of Genes Remaining
0	None - Raw Original Data	55628
1	Overlap-only Subset (removing ERCC spike-in protocol genes)	55536
2	Annotated pseudogenes filtering	20101
3	Sample coverage filtered based on Low Count	17772
4	Feature selection (mRMR) subsetting	60

Supplementary Table 2: Top 60 features selected from Harmonized Dataset using mRMR

Gene	Symbol
ENSMUSG00000058672	Tubb2a
ENSMUSG00000021556	Golm1
ENSMUSG00000112545	1300014J16Rik
ENSMUSG00000103864	Gm37415
ENSMUSG00000044068	Zrsr1
ENSMUSG00000033781	Asb13
ENSMUSG00000060509	Xcr1
ENSMUSG00000038393	Txnip
ENSMUSG00000049555	Tmie
ENSMUSG00000022324	Matn2
ENSMUSG00000026669	Mcm10
ENSMUSG00000033542	Arhgef5
ENSMUSG00000029287	Tgfbr3
ENSMUSG00000046962	Zbtb21
ENSMUSG00000050350	Gpr18
ENSMUSG00000026730	Pter
ENSMUSG00000066319	Rtp3
ENSMUSG00000019996	Map7
ENSMUSG00000042215	Bag2
ENSMUSG00000067787	Blcap
ENSMUSG00000034164	Emid1
ENSMUSG00000026179	Pnkd
ENSMUSG00000085715	Tsix
ENSMUSG00000056515	Rab31
ENSMUSG00000039067	Psmd7
ENSMUSG00000042406	Atf4
ENSMUSG00000027068	Dhrs9
ENSMUSG00000054843	Atrnl1
ENSMUSG00000030523	Trpm1
ENSMUSG00000050232	Cxcr3
ENSMUSG00000005547	Cyp2a5
ENSMUSG00000048521	Cxcr6
ENSMUSG00000037270	4932438A13Rik
ENSMUSG00000068011	Mkrn2os
ENSMUSG00000046916	Myct1
ENSMUSG00000027993	Trim2
ENSMUSG00000035266	Helq

ENSMUSG00000051969	Tlr11
ENSMUSG00000021918	Nek4
ENSMUSG00000036752	Tubb4b
ENSMUSG00000038884	A230050P20Rik
ENSMUSG00000069920	B3gnt9
ENSMUSG00000027698	Nceh1
ENSMUSG00000059183	Mtfmt
ENSMUSG00000074890	Lcmt2
ENSMUSG00000042496	Prdm10
ENSMUSG00000041538	H2-Ob
ENSMUSG00000016520	Lnx2
ENSMUSG00000067049	Unc93a
ENSMUSG00000029413	Naaa
ENSMUSG00000041119	Pde9a
ENSMUSG00000020182	Ddc
ENSMUSG00000035493	Tgfbi
ENSMUSG00000086669	AA645442
ENSMUSG00000036078	Sigmar1
ENSMUSG00000032026	Rexo2
ENSMUSG00000027478	Dnmt3b
ENSMUSG00000001630	Stk38l
ENSMUSG00000030255	Sspn
ENSMUSG00000021557	Agtpbp1

Supplementary Table 3: Gene Ontology Biological Processes Identified from SVM Merged-Study Analysis

ID	Description
GO:0002526	acute inflammatory response
GO:0006261	DNA-dependent DNA replication
GO:0022408	negative regulation of cell-cell adhesion
GO:0007162	negative regulation of cell adhesion
GO:0045342	MHC class II biosynthetic process
GO:0003009	skeletal muscle contraction
GO:0010951	negative regulation of endopeptidase activity
GO:0019884	antigen processing and presentation of exogenous antigen
GO:0045346	regulation of MHC class II biosynthetic process
GO:0000727	double-strand break repair via break-induced replication
GO:0031294	lymphocyte costimulation
GO:0009101	glycoprotein biosynthetic process
GO:0098659	inorganic cation import across plasma membrane
GO:0099587	inorganic ion import across plasma membrane
GO:0006271	DNA strand elongation involved in DNA replication
GO:0007600	sensory perception
GO:0006268	DNA unwinding involved in DNA replication
GO:0032400	melanosome localization
GO:0048305	immunoglobulin secretion
GO:0023061	signal release
GO:0018200	peptidyl-glutamic acid modification
GO:0007611	learning or memory
GO:0033619	membrane protein proteolysis
GO:0051875	pigment granule localization
GO:0002440	production of molecular mediator of immune response
GO:0050920	regulation of chemotaxis
GO:0031295	T cell costimulation
GO:0050879	multicellular organismal movement
GO:0050881	musculoskeletal movement
GO:0018095	protein polyglutamylation
GO:0099537	trans-synaptic signaling
GO:0006270	DNA replication initiation
GO:0019233	sensory perception of pain
GO:0052646	alditol phosphate metabolic process
GO:0031649	heat generation

GO:0050663	cytokine secretion
GO:0002827	positive regulation of T-helper 1 type immune response
GO:2000514	regulation of CD4-positive, alpha-beta T cell activation
GO:0032946	positive regulation of mononuclear cell proliferation
GO:0042060	wound healing
GO:0007268	chemical synaptic transmission
GO:0098916	anterograde trans-synaptic signaling
GO:0060055	angiogenesis involved in wound healing
GO:0046640	regulation of alpha-beta T cell proliferation
GO:0045620	negative regulation of lymphocyte differentiation
GO:0050890	cognition
GO:0006022	aminoglycan metabolic process
GO:0045785	positive regulation of cell adhesion
GO:1903510	mucopolysaccharide metabolic process
GO:0050731	positive regulation of peptidyl-tyrosine phosphorylation
GO:0045214	sarcomere organization
GO:0002347	response to tumor cell
GO:0032633	interleukin-4 production
GO:0006023	aminoglycan biosynthetic process
GO:0021694	cerebellar Purkinje cell layer formation
GO:0007379	segment specification
GO:1901890	positive regulation of cell junction assembly
GO:0030203	glycosaminoglycan metabolic process
GO:0035592	establishment of protein localization to extracellular region
GO:1901077	regulation of relaxation of muscle
GO:0002286	T cell activation involved in immune response
GO:0002418	immune response to tumor cell
GO:0032401	establishment of melanosome localization
GO:0043370	regulation of CD4-positive, alpha-beta T cell differentiation
GO:0006120	mitochondrial electron transport, NADH to ubiquinone
GO:0050921	positive regulation of chemotaxis
GO:0060338	regulation of type I interferon-mediated signaling pathway
GO:0007613	memory
GO:0045907	positive regulation of vasoconstriction
GO:0043501	skeletal muscle adaptation
GO:0051905	establishment of pigment granule localization
GO:0006638	neutral lipid metabolic process

GO:0006639	acylglycerol metabolic process
GO:0008015	blood circulation
GO:0001660	fever generation
GO:0007601	visual perception
GO:0032945	negative regulation of mononuclear cell proliferation
GO:2000516	positive regulation of CD4-positive, alpha-beta T cell activation
GO:0009611	response to wounding
GO:0006953	acute-phase response
GO:0021692	cerebellar Purkinje cell layer morphogenesis
GO:0071692	protein localization to extracellular region
GO:0030239	myofibril assembly
GO:0032402	melanosome transport
GO:0055001	muscle cell development
GO:0002834	regulation of response to tumor cell
GO:0002837	regulation of immune response to tumor cell
GO:0002790	peptide secretion
GO:0099054	presynapse assembly
GO:2000379	positive regulation of reactive oxygen species metabolic process
GO:0060009	Sertoli cell development
GO:0050707	regulation of cytokine secretion
GO:0043405	regulation of MAP kinase activity
GO:0099177	regulation of trans-synaptic signaling
GO:0061383	trabecula morphogenesis
GO:0035994	response to muscle stretch
GO:0007610	behavior

Supplementary Table 4: Top genes identified from the SVM merged-study gene set enrichment analysis

ENSEMBLID	SYMBOL
ENSMUSG00000027398	Il1b
ENSMUSG00000002603	Tgfb1
ENSMUSG00000035283	Adrb1
ENSMUSG00000049103	Ccr2
ENSMUSG00000045730	Adrb2
ENSMUSG00000020122	Egfr
ENSMUSG00000026981	Il1rn
ENSMUSG00000031132	Cd40lg
ENSMUSG00000022892	App
ENSMUSG00000026573	Xcl1
ENSMUSG00000031778	Cx3cl1
ENSMUSG00000020484	Xbp1
ENSMUSG00000037944	Ccr7
ENSMUSG00000027950	Chrnb2
ENSMUSG00000004110	Cacna1e
ENSMUSG00000024109	Nrxn1
ENSMUSG00000079037	Prnp
ENSMUSG00000019969	Psen1
ENSMUSG00000035042	Ccl5
ENSMUSG00000016496	Cd274
ENSMUSG00000062960	Kdr
ENSMUSG00000027239	Mdk
ENSMUSG00000020716	Nf1
ENSMUSG00000005952	Trpv1
ENSMUSG00000024597	Slc12a2
ENSMUSG00000024261	Syt4
ENSMUSG00000031012	Cask
ENSMUSG00000007655	Cav1
ENSMUSG00000061731	Ext1
ENSMUSG00000024610	Cd74
ENSMUSG00000004296	Il12b
ENSMUSG00000020399	Havcr2
ENSMUSG00000027859	Ngf
ENSMUSG00000027765	P2ry1
ENSMUSG00000026778	Prkcq
ENSMUSG00000031840	Rab3a

ENSMUSG00000033860	Fgg
ENSMUSG00000079055	Slc8a3
ENSMUSG00000025888	Casp1
ENSMUSG00000034987	Hrh2
ENSMUSG00000033540	Idua
ENSMUSG00000018899	Irf1
ENSMUSG00000055254	Ntrk2
ENSMUSG00000020077	Srgn
ENSMUSG00000041135	Ripk2
ENSMUSG00000049313	Sorl1
ENSMUSG00000071005	Ccl19

Supplementary Table 5: Overlapping significant gene ontology (GO) biological processes (BP) from GSEA between single-study analysis of RR1 (NASA), RR6, RR8, RR9, and merged-study SVM analysis

GO ID	Parent GO ID	Term Description	Parent Term
GO:0002250	GO:0002250	adaptive immune response	adaptive immune response
GO:0060326	GO:0060326	cell chemotaxis	cell chemotaxis
GO:0006959	GO:0002250	humoral immune response	adaptive immune response
GO:0032543	GO:0032543	mitochondrial translation	mitochondrial translation
GO:0019882	GO:0002250	antigen processing and presentation	adaptive immune response
GO:0022409	GO:0022409	positive regulation of cell-cell adhesion	positive regulation of cell-cell adhesion
GO:0050870	GO:0022409	positive regulation of T cell activation	positive regulation of cell-cell adhesion
GO:0050900	GO:0060326	leukocyte migration	cell chemotaxis
GO:0050778	GO:0002250	positive regulation of immune response	adaptive immune response
GO:0002821	GO:0002250	positive regulation of adaptive immune response	adaptive immune response
GO:0002819	GO:0002250	regulation of adaptive immune response	adaptive immune response
GO:0002768	GO:0002250	immune response-regulating cell surface receptor signaling pathway	adaptive immune response
GO:1903039	GO:0022409	positive regulation of leukocyte cell-cell adhesion	positive regulation of cell-cell adhesion
GO:0002253	GO:0002250	activation of immune response	adaptive immune response
GO:0002443	GO:0002250	leukocyte mediated immunity	adaptive immune response
GO:0002460	GO:0002250	adaptive immune response based on somatic recombination of immune receptors built from immunoglobulin superfamily domains	adaptive immune response
GO:0001906	GO:0001906	cell killing	cell killing
GO:0002709	GO:0002250	regulation of T cell mediated immunity	adaptive immune response

GO:0002822	GO:0002250	regulation of adaptive immune response based on somatic recombination of immune receptors built from immunoglobulin superfamily domains	adaptive immune response
GO:0002824	GO:0002250	positive regulation of adaptive immune response based on somatic recombination of immune receptors built from immunoglobulin superfamily domains	adaptive immune response
GO:0031347	GO:0031347	regulation of defense response	regulation of defense response
GO:0032103	GO:0031347	positive regulation of response to external stimulus	regulation of defense response
GO:0002449	GO:0002250	lymphocyte mediated immunity	adaptive immune response
GO:0031349	GO:0031347	positive regulation of defense response	regulation of defense response
