# Supplementary material for: Harmonizing heterogeneous transcriptomics datasets for machine learning-based analysis to identify spaceflown murine liver-specific changes
Source: NPJ Microgravity. 2024 Jun 11;10:61. doi: 10.1038/s41526-024-00379-3 (PMC11167036; doi:10.1038/s41526-024-00379-3)
Supplement: Supplementary file 2 — Reporting Summary [file 41526_2024_379_MOESM2_ESM.pdf]

nature portfolio

Corresponding author(s): Hari Ilangovan

Last updated by author(s): Feb 22, 2024

# Reporting Summary

Nature Portfolio wishes to improve the reproducibility of the work that we publish. This form provides structure for consistency and transparency in reporting. For further information on Nature Portfolio policies, see our Editorial Policies and the Editorial Policy Checklist.

## Statistics

For all statistical analyses, confirm that the following items are present in the figure legend, table legend, main text, or Methods section.

| n/a | Confirmed | |
|---|---|---|
| ☐ | ☒ | The exact sample size (*n*) for each experimental group/condition, given as a discrete number and unit of measurement |
| ☐ | ☒ | A statement on whether measurements were taken from distinct samples or whether the same sample was measured repeatedly |
| ☐ | ☒ | The statistical test(s) used AND whether they are one- or two-sided<br>*Only common tests should be described solely by name; describe more complex techniques in the Methods section.* |
| ☐ | ☒ | A description of all covariates tested |
| ☐ | ☒ | A description of any assumptions or corrections, such as tests of normality and adjustment for multiple comparisons |
| ☐ | ☒ | A full description of the statistical parameters including central tendency (e.g. means) or other basic estimates (e.g. regression coefficient) AND variation (e.g. standard deviation) or associated estimates of uncertainty (e.g. confidence intervals) |
| ☐ | ☒ | For null hypothesis testing, the test statistic (e.g. *F*, *t*, *r*) with confidence intervals, effect sizes, degrees of freedom and *P* value noted<br>*Give P values as exact values whenever suitable.* |
| ☒ | ☐ | For Bayesian analysis, information on the choice of priors and Markov chain Monte Carlo settings |
| ☒ | ☐ | For hierarchical and complex designs, identification of the appropriate level for tests and full reporting of outcomes |
| ☒ | ☐ | Estimates of effect sizes (e.g. Cohen's *d*, Pearson's *r*), indicating how they were calculated |

*Our web collection on statistics for biologists contains articles on many of the points above.*

## Software and code

Policy information about availability of computer code

| | |
|---|---|
| Data collection | No software was used for data collection in this study. All data were publicly available on NASA's Open Science Data Repository. |
| Data analysis | Custom code, available on NASA's Github account under the trrac repository, is used to analyse the data in this study. All code is comprised of open source R and Python packages that are free for research purposes. Details on the component packages can be found at the aforementioned repository. |

For manuscripts utilizing custom algorithms or software that are central to the research but not yet described in published literature, software must be made available to editors and reviewers. We strongly encourage code deposition in a community repository (e.g. GitHub). See the Nature Portfolio guidelines for submitting code & software for further information.

## Data

Policy information about availability of data

All manuscripts must include a data availability statement. This statement should provide the following information, where applicable:

- Accession codes, unique identifiers, or web links for publicly available datasets
- A description of any restrictions on data availability
- For clinical datasets or third party data, please ensure that the statement adheres to our policy

The datasets analysed in this study are publicly available RNA-seq counts data and metadata on NASA’s Open Science Data Repository (OSDR) under the OSDR-47 ,

-168, -242, -245, and -379 database entries. The harmonized datasets generated in this study can be replicated from the code available on NASA's Github account under the trrac repository at https://github.com/nasa/trrac.

## Research involving human participants, their data, or biological material

Policy information about studies with human participants or human data. See also policy information about sex, gender (identity/presentation), and sexual orientation and race, ethnicity and racism.

| | |
|---|---|
| Reporting on sex and gender | N/A - no human subjects |
| Reporting on race, ethnicity, or other socially relevant groupings | N/A - no human subjects |
| Population characteristics | N/A - no human subjects |
| Recruitment | N/A - no human subjects |
| Ethics oversight | N/A - No human subjects |

Note that full information on the approval of the study protocol must also be provided in the manuscript.

# Field-specific reporting

Please select the one below that is the best fit for your research. If you are not sure, read the appropriate sections before making your selection.

☒ Life sciences ☐ Behavioural & social sciences ☐ Ecological, evolutionary & environmental sciences

For a reference copy of the document with all sections, see nature.com/documents/nr-reporting-summary-flat.pdf

# Life sciences study design

All studies must disclose on these points even when the disclosure is negative.

| | |
|---|---|
| Sample size | All available transcriptomics data from the Rodent Research (RR) 1 NASA, RR-1 CASIS, RR-3, RR-6, RR-8, and RR-9 missions were used. This resulted in a total of 153 murine subjects across all studies. The purpose of this study was to use all conducted missions, so the authors in this study were not involved in sample-size calculations. In our study, we explain in detail how we validate that the sample sizes were sufficient for our modeling approaches. |
| Data exclusions | Murine samples were excluded from analysis upon identification of outliers (n=16). Outliers were determined from principal component analysis, distance from treatment group clusters, and RNA quality from transcriptomics profiling. |
| Replication | We use all publicly available data comes from six separate Rodent Research missions, and we utilized 5-fold cross validation in our training and testing methodology. |
| Randomization | A training and testing split was applied to the data and balanced across the treatment groups. The samples were randomized during training using a stratified 5-fold cross validation procedure, which was repeated multiple times to get an average performance and ensure randomization. |
| Blinding | Blinding was not applicable to this analysis. |

# Reporting for specific materials, systems and methods

We require information from authors about some types of materials, experimental systems and methods used in many studies. Here, indicate whether each material, system or method listed is relevant to your study. If you are not sure if a list item applies to your research, read the appropriate section before selecting a response.

## Materials & experimental systems

| n/a | Involved in the study | |
|---|---|---|
| ☒ | ☐ | Antibodies |
| ☒ | ☐ | Eukaryotic cell lines |
| ☒ | ☐ | Palaeontology and archaeology |
| ☐ | ☒ | Animals and other organisms |
| ☒ | ☐ | Clinical data |
| ☒ | ☐ | Dual use research of concern |
| ☒ | ☐ | Plants |

## Methods

| n/a | Involved in the study | |
|---|---|---|
| ☒ | ☐ | ChIP-seq |
| ☒ | ☐ | Flow cytometry |
| ☒ | ☐ | MRI-based neuroimaging |

# Animals and other research organisms

Policy information about studies involving animals; ARRIVE guidelines recommended for reporting animal research, and Sex and Gender in Research

Laboratory animals: The mus musculus subjects in this study included C57BL/6NTac strain from RR-1 CASIS (32 wks) and RR-6 (32 wks), C57BL/6J strain from RR-1 NASA (16 wks) and RR-9 (10 wks), BALB/cAnNTac train from RR-8 (10-32 wks), and BALB/c strain from RR-3 (12 wks). All ages are reported as ages at launch.

Wild animals: N/A

Reporting on sex: The analysis from this study include male and female murine subjects. The model training procedure is stratified by sex to preserve sex distributions in the training and testing sets.

Field-collected samples: N/A

Ethics oversight: This is secondary use of publicly available data from Open Science Data Repository. All rodent research missions conducted through NASA and executed on the international space station were overseen by the NASA Human Research Program. All ground based controls were overseen by academic partners that have been funded by NASA.

Note that full information on the approval of the study protocol must also be provided in the manuscript.

# Plants

Seed stocks: N/A

Novel plant genotypes: N/A

Authentication: N/A
